# Silica Aerogels in Nano Drug Delivery Systems: A Comprehensive Review from Preparation to Medical Applications

**DOI:** 10.3390/gels11110859

**Published:** 2025-10-27

**Authors:** Xinran Qian, Jialu Lu, Meili Rui, Dengyun Xu, Haohan Liu, Dongxiao Han, Tianfeng Lu, Jianming Yang, Ai Du, Lili Qin

**Affiliations:** 1 Sports and Health Research Center, Department of Physical Education, Tongji University, Shanghai 200092, China; 2 Shanghai Key Laboratory of Special Artificial Microstructure Materials and Technology, School of Physics Science and Engineering, Tongji University, Shanghai 200092, China

**Keywords:** silica aerogel, drug delivery systems, nanomedicine, medical applications

## Abstract

Silica aerogel has garnered significant attention in the biomedical field, primarily due to its unique combination of a three-dimensional structure, low density, tunable nanoscale pores, and an extensive surface area. These intrinsic properties render it as an exceptional candidate for advanced drug delivery systems (DDSs). In the realm of medical applications, silica aerogels have demonstrated remarkable potential, especially in nanoscale DDSs. Traditional drug delivery methods, such as capsules and tablets, are often plagued by several drawbacks, including poor bioavailability, lack of target specificity, and multidrug resistance. These limitations necessitate the development of more efficient and targeted drug delivery systems. Recent advancements in the synthesis and modification of silica aerogels have significantly enhanced their biocompatibility and functionalization capabilities. These improvements have further bolstered their potential for controlled release and targeted delivery of therapeutic agents. This study is based on silica aerogel-based nanocarrier systems, providing an in-depth exploration of its fundamental principles, preparation processes, and recent advancements. Based on this, we summarize the drug delivery methods, drug release characteristics, and diverse medical applications of silica aerogels. Additionally, we discuss the challenges and future prospects of applying silica aerogels in drug delivery systems, aiming to provide a comprehensive overview of this field.

## 1. Introduction

Drug delivery systems (DDSs) have received an increasing amount of attention in the field of biomedical research. DDSs are defined as a formulation or device that improves bioavailability by preventing premature degradation and enhancing the uptake of active ingredients [1]. As a commercially available process, DDSs have benefited tens of millions of patients by relieving suffering and prolonging life from diverse diseases [2]. The advancement of nanotechnology has also facilitated the study and development of DDSs. With the development and progress of nanotechnology and the advantages of nanoparticles, such as reliable biocompatibility and biodegradability, DDSs have been widely discussed and applied. The drugs are loaded inside or modified on the surface of the nanoparticles to achieve the properties that the pure drugs do not have, thus the application of nanoparticle drug delivery systems (NPDDSs) in therapy can solve the problems found in a traditional drug therapy benefiting from the physicochemical properties of the nanocarriers. As the particle size is much smaller than that of the capillary pathway, with the significant advantages of improving drug stability, facilitating the crossing of biofilms, reducing drug toxicity and side effects, controlling the drug release rate, and targeting to disease site and target cells, DDSs have been widely used in biomedical field [3,4,5].

Nowadays, aerogel material has become a new drug delivery system strategy owing to its simple structure, easy-to-modify moieties, lightweightness, high surface areas, and porous structure. In recent years, a variety of nanocarriers have been developed for drug delivery, including liposomes, polymeric nanoparticles, dendrimers, and solid lipid nanoparticles, which offer improved solubility, stability, and controlled release of therapeutic agents [5]. Among these, silica-based nanomaterials have attracted particular attention due to their tunable porosity, large surface area, and biocompatibility. Since Kistler’s first discovery in the 1930s, silica aerogel has been widely discussed because of its considerable potential for applications, such as adsorption [6], thermal and acoustic insulation [7,8], catalysis [9], benefiting from its high porosity (80–99.8%), high specific surface area (500–1200 m^2^/g), low density (0.003–0.5 g/cm^3^), low thermal conductivity (0.005–0.1 W/m-K), dielectric constant, refractive index, and other outstanding features [10]. Importantly, these features arise from a nanoporous network structure composed of silica nanoparticles interconnected at the nanometer scale (10–100 nm), endowing the material with typical characteristics of nanomaterials. There is also a strong focus on biomedical research, including drug delivery, tissue support, implantable materials, bioimaging and biosensing [11]. Owing to these unique nanoscale characteristics, silica aerogels are highly suitable nanocarriers for efficient, targeted, and safe drug delivery applications. Silica aerogel can be adjusted by manipulating the synthesis conditions and hybridizing with other materials when loaded with drugs, active ingredients, enzymes, and proteins, becoming one of the most promising carriers in NPDDSs.

With silica aerogel materials stepping into the industrial application stage, and the maturation of the process, silica aerogel has a high output and high commercial demands, and has been widely applied in food supplements, medical and other fields [12]. In addition to traditional applications, silica aerogels have been successfully employed to encapsulate poorly water-soluble drugs and proteins, exhibiting higher loading capacities and more precise controlled-release profiles compared with other nanocarriers, including liposomes and polymeric nanoparticles. The physicochemical characterization of aerogels is sufficient for other applications, but for biomedical applications, biocompatibility must be tested by in vivo and in vitro methods [13]. In humans, silicon is an essential micronutrient, and plays an important role in bone and connective tissue health [14]. Silica aerogel as a drug delivery system has been proved to be biocompatible, featuring biodegradability, cytotoxicity, pharmacokinetics, and histocompatibility [15,16,17,18], and can enhance the dissolution rate of poorly water-soluble drugs [19,20]. Silica is the most abundant mineral in the Earth’s crust (~75 wt%) [21], representing the natural resources in the continental and ocean plates. Multiple forms of silica have been used in a wide range of nanotechnological applications. Except from mineralogical rocks, such as quartzite, silica can also be found from agricultural wastes [22,23]. Knowing that rice husk ash contains 70–98 wt% of silica, Rajanna et al. extracted robust silica aerogel microspheres using the sol–gel/emulsion method [21]. The resultant aerogel boasts exceptional bioactivity and cell viability characteristics, rendering it an ideal material for applications in tissue engineering.

Current research on DDSs mainly focuses on continuing to explore new polymers, stimulus–response mechanisms, and other areas [24]. The unique properties of silica aerogels, such as their high surface area, porosity, and biocompatibility, make them ideal for enhancing drug stability, reducing drug toxicity and side effects, controlling drug release rates, and targeting specific disease sites and cells [25]. This paper provides a comprehensive review of the preparation methods and medical applications of silica aerogel drug delivery systems, with a focus on the transformative potential of silica aerogel in revolutionizing the field of drug delivery. It highlights its ability to break through traditional treatment models, offering the potential to transform how we approach disease treatment in the future and opening new avenues for medical innovation. In this study, we systematically evaluate the advantages and limitations of major drug-loading routes, including sol–gel incorporation, supercritical CO_2_ adsorption, solvent-based impregnation, and surface functionalization, thereby identifying the most promising strategies for stable and efficient drug delivery. Additionally, we include a dedicated discussion on regulatory and translational considerations, addressing approval pathways, safety standards, and challenges that must be overcome for clinical implementation. Collectively, these perspectives aim to bridge fundamental materials research with biomedical application and translational medicine.

## 2. Preparation and Drug Loading of Silica Aerogel

### 2.1. Preparation of Silica Aerogel

**Figure 1:**
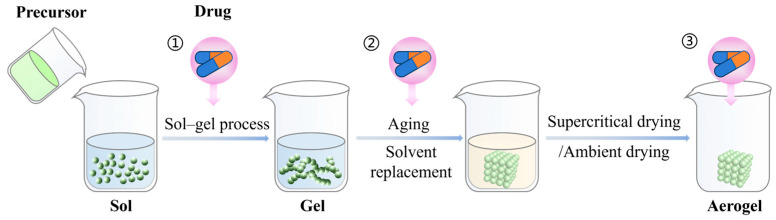
Preparation process of drug-loaded silica aerogel.

The common preparation process of silica aerogel is the sol–gel method, usually prepared by two-step acid–base catalysis. The preparation process of silica aerogel for DDSs at the present stage can be briefly summarized as the sol–gel process, aging, drying, etc. Each step requires the modulation of relevant parameters to adjust the properties of the aerogel structure, such as concentration of precursors, pH, temperature, solvent, and time, among others. Figure 1 illustrates the common preparation process of drug-loaded silica aerogel.

#### 2.1.1. Precursor Selection and Gel Formation

The precursor can be a water-based sodium silicate (water glass) or an organosilicon source. Tetraethyl orthosilicate (TEOS) and methyl orthosilicate (TMOS) are mainly used as organosilicon silicone raw materials [26]. The hydrolysis of the silicon source is carried out under acidic conditions and requires base-induced Si–OH condensation upon completion. The silica molecules in the solution system continue to combine and form a three-dimensional network structure. As the reaction proceeds, the sol eventually transforms into a gel [27]. In the gel, the solvent is mostly water, leading to a high surface tension, which can cause the nanostructures to collapse because of capillary forces during the drying process. Therefore, during solvent replacement, it is necessary to modify the wet gel by means of low surface tension solvents, such as alcohols or alkanes. After aging, the mechanical strength of the wet gel is improved.

#### 2.1.2. Drying Methods and Their Characteristics

The appropriate drying method will affect the characteristics of the final product. The main drying methods include the supercritical drying method, the freeze-drying method, and the ambient pressure drying method. Usually, silica aerogels are prepared by supercritical drying; the solvent in the wet gel is converted into a supercritical state without surface tension [10]. This method maintains the structural integrity of the material, and allows for a relatively fast drying process. In the freeze-drying method, the wet gel is first frozen, and then the solvent is removed by sublimation under vacuum. This process helps to maintain the porous structure of the aerogel without the need for high pressure, making it a cost-effective and environmentally friendly option [28]. However, due to the cost of the freeze-drying process, ambient pressure drying has become a safe and inexpensive technology for industrial production [29], but it often leads to partial shrinkage and reduced pore volume unless additional surface modification or precursor optimization is introduced.

Supercritical drying remains the benchmark method for achieving highly porous aerogels with minimal structural collapse; however, the high operational cost and reliance on specialized high-pressure equipment greatly limit its feasibility for large-scale or clinical production. Freeze-drying provides a milder alternative that avoids extreme conditions and can preserve delicate pore networks, yet its long processing time and high energy consumption make it less practical for industrial translation. Overall, while supercritical drying is best suited for high-quality research-grade materials, ambient pressure drying—with further refinement—appears to be the most promising strategy for the clinical and industrial deployment of silica aerogels.

#### 2.1.3. Recent Advances in Preparation Methods

The preparation methods of silica aerogel have also been updated. Using inorganic silicone raw materials, such as water. glass. and n-hexane emulsion, spherical silica aerogel powders with narrow particle size distribution can be synthesized by polymerization, which can significantly reduce the production time [30]. Zhang et al. designed a simple method without applying any surfactants to synthesize silica aerogel microspheres via the atmospheric pressure drying process, packing density 62 mg/cm^3^ to 230 mg/cm^3^. The high BET surface area and hierarchically porous structure have potential in the loading of drugs [31]. Silica aerogel from natural resources and waste materials is low-cost and environmentally friendly [32]. Rice husk, an agricultural waste from rice production, is a renewable source with biomass components, including cellulose, lignin, and silica. Rice husk burning produces rice husk ash containing a large amount of silica (85–90%). Cheap silica aerogels have been synthesized from rice husk ash using a simple sol–gel aqueous ambient pressure drying (APD) technique. The use of rice husk ash as a source of silica aerogel is of great interest in the biomedical field [19,33,34,35].

### 2.2. Drug-Loaded Silica Aerogel

The loading of drugs into silica aerogels can be achieved through various stages of the aerogel production process, each offering unique advantages and suitable for different types of drugs. In summary, the process of drug loading into the aerogel can be simply summarized as the addition of the drug in the sol–gel process, during the aging process, and in the dried aerogel. Table 1 presents the processes and methods for loading different drugs into silica aerogels. Meanwhile, in Table 2, we have listed the types of drugs loaded into silica aerogels and their corresponding methods for reference.

#### 2.2.1. Sol–Gel Process

Generally, drugs added into the sol–gel process before gelation is a straightforward and commonly used drug-loading method. The addition of drugs or other functional compounds during the sol–gel transition has been shown to enhance drug encapsulation within the aerogel matrix [36]. This method is particularly advantageous for drugs which dissolve well in the liquid phase of the precursor solution. In this way, it is possible to obtain materials where the drug is chemically or physically bound to the solid phase [37,38]. For instance, dispersing resveratrol in the hydrolyzed TEOS ethanol solution, Qin et al. designed resveratrol-loaded silica aerogel (RSA) as a drug delivery system with a high loading rate of 19%. RSA successfully improved the performance of pure resveratrol, and showed a sustained releasing effect [16].

#### 2.2.2. Aging Process

The aging process enhances the network structure, pore structure, and mechanical properties of silica sol. After gelation, drugs can be added to the aging solution, allowing them to diffuse into the pores of the gel. Drugs which can dissolve in aging solutions and remain stable during the aging process can be added to the aging solvents at this stage. This period provides an opportunity for drug molecules to further integrate into the gel network, potentially improving the loading efficiency. Wang et al. prepared trans-resveratrol (RSV)-loaded silica aerogel (RLSA), adding the drug during the aging process. The drug diffused into the aerogel pores from the aging solution containing the RSV. They also established a fundamental model to find that RSV stays in the silica aerogel in two ways and to study the interaction of diffusion and adsorption/desorption [39].

#### 2.2.3. In the Dried Aerogel

Loading drugs into the dried silica aerogels is a highly effective method, which is often referred to as post-drying modification or adsorption precipitation. Impregnating drugs into pre-formed aerogels can enhance drug loading capacities and improve release profiles.

##### Supercritical CO_2_ Adsorption

Supercritical CO_2_ (scCO_2_) adsorption is the most commonly used method. The use of a non-toxic solvent (CO_2_) as a supercritical liquid observes the principles of “green” chemistry, which is important in DDSs. In addition, appropriate parameters for supercritical adsorption, including temperature, pressure, and time, can be selected in accordance with the stability of the drug [40]. Adsorption occurs in the scCO_2_ process, causing poorly soluble drugs to be distributed at the molecular level within the silica aerogel matrix, which can significantly alter the drug release rate [41].

Mohammadian et al. used the supercritical deposition method to make the model drug ketoprofen (a low water-soluble drug) absorbed into the pores of the aerogel uniformly, and enhanced the solubility and release of the drug with an amorphous structure. After cytotoxicity testing, the silica aerogel exhibited a high potential for cell growth and cell survival, without exhibiting cytotoxicity [42]. Moreover, rapid depressurization during the scCO_2_ loading process rate can lead to high supersaturation levels, promoting the precipitation of additional drug molecules within the aerogel pores [43]. Singh et al. selected benzoic acid (BA) and fenofibrate (FF) with different molecular mass and solubilities in water and supercritical carbon dioxide as drugs for a comparison of the loadings by the two modes [44].

##### Solvent-Based Methods

Solvent-based methods are suitable for drugs that are soluble in the chosen solvent and can achieve high drug-loading capacities and controlled release profiles. These methods allow the drug molecules to rearrange and aggregate inside the aerogel pores, resulting in improved release kinetics. After the drug loading process, the solvent must be removed to the acceptable level specified in the International Conference on Harmonization (ICH) Q3 (R5) guidelines [45]. The main challenges in using the solvent method for loading lie in the concentration of the drug used, the selection of an appropriate solvent, and the regulation of the filling factor.

The solvent evaporation method involves dispersing the aerogel in a drug solution and drying it by rapid solvent evaporation [46]. The drug dissolves in an appropriate solvent to form a uniform drug solution, and then the silica aerogel is immersed in the drug solution, allowing the drug molecules to enter the pores of the aerogel through capillary action and become fixed within the pores [47]. Mellaerts et al. used the solvent evaporation method to load itraconazole and ibuprofen into SBA-15 silica [48]. They observed that the drug molecules were positioned inside the micropores, resulting in faster release kinetics compared to other methods.

Diffusion supported loading (DiSupLo) is a new extremely easy and efficient method for loading drugs. Katarzyna Trzeciak et al. [49] first proposed this method for drug delivery using mesoporous silica nanoparticles (MSNs). In the study, the pre-homogenized mixture of drugs and MSNs was placed in a sealed container containing ethanol and left to stand, thereby achieving high filling factor and complete encapsulation of the pharmaceutical ingredients. Elif Çalışkan Salihi et al. [50] added curcumin or methylene blue to the aqueous dispersion of aerogel samples. Drug loading was achieved after shaking for 24 h, and the samples were collected by centrifugation. This method is expected to be used in the future for the preparation of silica aerogel DDSs, enabling simple and efficient drug loading.

##### Surface Functionalization

**Figure 2:**
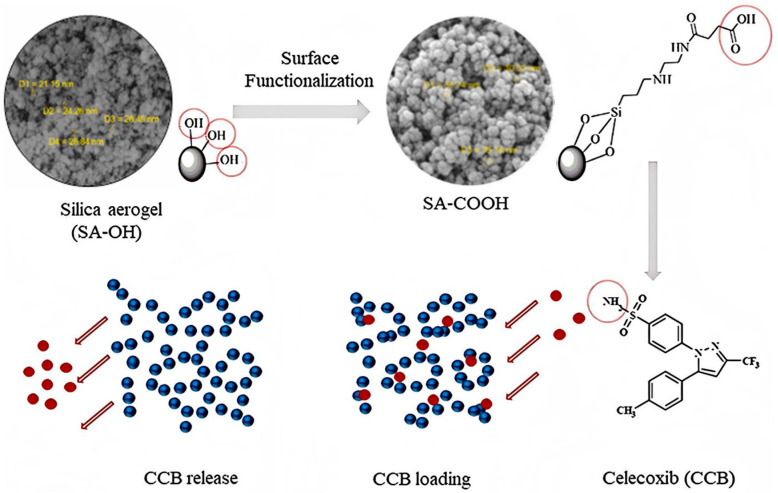
Silica aerogel surface functionalization loaded with carboxylic acid. Reproduced with permission from Elsevier, Reference [52].

Post-drying modifications offer a final opportunity to incorporate drugs into the aerogel structure. Surface functionalization introduces specific functional groups onto the surface of the silica aerogel through chemical modification [51]. This enhances the interaction between the drug and the aerogel, thereby improving drug loading capacity and release performance. On the surface of two-step carboxyl-modified silica aerogels, the model drug celecoxib can be bonded between sulfonamide groups, showing that carboxyl-functionalized silica aerogels can improve the water solubility of some poorly water-soluble drugs and control their dissolution rates [52]. Figure 2 illustrates the process of carboxylic acid loading onto the surface of silica aerogel. In addition, hydrophobic surface modification not only enhances the moisture resistance of aerogels but improves their long-term stability and functionality [53]. For example, surface modification using trimethylchlorosilane (TMCS) can prevent the collapse of the aerogel structure during drying by reducing capillary forces [54], thereby better maintaining the porous structure for the loading of drug molecules. Appropriate surface functionalization strategies can further optimize the drug loading capacity and release the characteristics of aerogels, improving the performance of silica aerogels in drug delivery systems and meeting the needs of different medical applications.

**Table 2:** Drug loading method and drug type.

Loading Process	Drug Loaded	Loading Method	Types of Drugs	Applications	References
sol–gel process					
	resveratrol	precursor solution	anti-inflammatory drug	bone/tissue reparation	[16]
aging process					
	resveratrol	RSV–EtOH solution	anti-inflammatory drug	bone/tissue reparation	[39]
	chlorhexidine	chlorhexidine digluconate	antibacterial drug	bone/tissue reparation	[55]
in the dried aerogel					
	camptothecin	solvent-based methods	anti-tumor drug	cancer therapy	[56]
	cinnamaldehyde	scCO_2_ adsorption	antibacterial drug	bone/tissue reparation	[57]
	salicylic acid	scCO_2_ adsorption	antibacterial drug	inflammatory therapy transdermal therapy	[57]
	sorbic acid	scCO_2_ adsorption	antibacterial drug	pharmaceutical preservation	[57]
	nimesulide	scCO_2_ adsorption	anti-inflammatory drug	inflammatory therapy	[20]
	ketoprofen	scCO_2_ adsorption	anti-inflammatory drug	transdermal therapy	[42]
	paclitaxel	solvent-based methods	anti-tumor drug	cancer therapy	[25]
	5-fluorouracil	solvent-based methods	anti-tumor drug	cancer therapy	[58]
	dithranol	scCO_2_ adsorption	anti-psoriasis drug	transdermal therapy cancer therapy	[59]
	doxorubicin	solvent-based methods	anti-tumor drug	cancer therapy photothermal therapy	[60]
	celecoxib	surface functionalization	anti-inflammatory drug	bone/tissue reparation	[61]
	fluconazole	solvent-based methods	antibacterial drug	transdermal therapy	[62]
	curcumin	solvent-based methods	anti-inflammatory drug	bone/tissue reparation photothermal therapy	[63]
	griseofulvin	scCO_2_ adsorption	antibacterial drug	transdermal therapy	[64]
	terfenadine	scCO_2_ adsorption	antihistamines	transdermal therapy	[64]
	niclosamide	scCO_2_ adsorption	antiparasitic drug	cancer therapy	[64]
	miconazole	scCO_2_ adsorption	antibacterial drug	transdermal therapy	[65]
	triflusal	scCO_2_ adsorption	antiplatelet drug	cancer therapy	[66]
	flurbiprofen	scCO_2_ adsorption	anti-inflammatory drug	transdermal therapy	[41]
	ibuprofen	scCO_2_ adsorption	anti-inflammatory drug	transdermal therapy	[41]
	fenofibrate	scCO_2_ adsorption/ solvent evaporation	lipid-lowering drug	cancer therapy	[44,48]
	benzoic acid	scCO_2_ adsorption	antibacterial drug	transdermal therapy	[44]
	itraconazole	solvent evaporation	antibacterial drug	transdermal therapy	[48]

## 3. Drug Release in Silica Aerogel Nanoparticles Drug Delivery System

### 3.1. Advantages of Silica Aerogel as a Drug Carrier

Among the common routes of administration, such as oral and injectable systemic methods, the administration is typically systemic and requires large quantities, which can harm other organs and may not be effective. Sometimes, due to the severity of the disease or the inherent toxicity of the drug, it must be applied directly to the target organ. Controlling release could improve drug utilization and reduce drug side effects. By adjusting the composition of the nanoparticle system, drug release can also be controlled and maintained at therapeutic levels. Drug release studies have shown that a high drug loading capacity can be achieved using aerogel as a carrier system. Silica aerogel was reported as a promising material, which can be used to promote treatment by incorporating active ingredients and drugs [67]. For example, by comparing the drug loading and release of silica aerogel and PVA nano fibers, drug-loaded silica aerogel was more effective due to its porosity and mesoporous structure [68]. In order to inhibit tumor growth, Wang et al. developed a nanoporous silica aerogel drug delivery system for oral paclitaxel that improves bioavailability and reduces drug side effects [25]. Usually, the drugs can be deposited in an amorphous state rather than in a crystalline form on the large surface area of the aerogel, which facilitates rapid dissolution and release in the body after oral administration and enables stimulus-responsive drug release.

### 3.2. Application of Composite Silica Aerogels in Drug Release

Composite aerogels combining the advantages of materials can optimize the properties of pure silica aerogels for drug delivery, such as controlled release kinetics. The addition of polymers changes the composition of the silica backbone to form Si–C bonds instead of the Si–O–Si bonds of pure silica aerogels, which enhances mechanical properties and fits to produce promising carriers. Organic/inorganic hybrid aerogels are gaining attention in the field, allowing for better adjustment of drug release from the silica aerogel matrix [69,70]. For example, silica–gelatin composite aerogels are used as novel DDSs for the controlled release of drugs. Veres et al. studied the relationship between matrix structure and release kinetics of the silica–gelatin aerogels (gelatin content of 3 wt%) and silica aerogels, and concluded that the strong hydration of the silica–gelatin skeleton promotes rapid desorption and dissolution of the drug from the loaded aerogel [71]. The gelatin content could control the structure of the silica–gelatin composite aerogels, which with low gelatin content (4–11 wt%) release the loaded drug rapidly, showing an initial burst; aerogels with a high gelatin content (18–24 wt%) have a significantly slower release and show retarded characteristics [72].

Additionally, core–shell structured particles are commonly used for drug delivery applications. Giray et al. developed a silica drug delivery system with a PEG hydrogel encapsulated as potential drug delivery carriers to achieve the sequential release of drugs with different hydrophobicity. The release profile of a drug (ketoprofen) can be controlled by adjusting the hydrophobicity of the aerogel core and the permeability of the PEG hydrogel shell [73]. With new emulsification technique, three different coated and uncoated forms of silica–alginate beads were prepared. Anticancer drugs were incorporated into the beads through emulsification and internal solidification technology, resulting in sustained and burst release [74].

## 4. Drug-Loaded Silica Aerogels in Medical Applications

**Figure 3:**
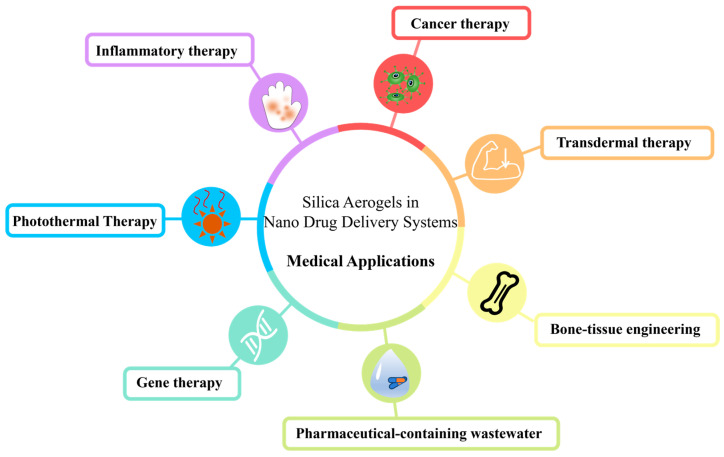
Drug-loaded silica aerogel in medical applications.

The unique functionality of silica aerogels and mature preparation processes open up new possibilities for advanced drug delivery systems. Disease-specific needs and drug properties can be settled through modifying the aerogel and compounding with other materials. A series of studies have also been conducted in various applications for the treatment of diseases. As shown in Figure 3, we have categorized the applications of drug-loaded silica aerogels into seven directions: inflammatory therapy, cancer therapy, transdermal therapy, bone/tissue reparation, photothermal therapy, gene therapy, and pharmaceutical-containing wastewater.

### 4.1. Inflammatory Therapy

**Figure 4:**
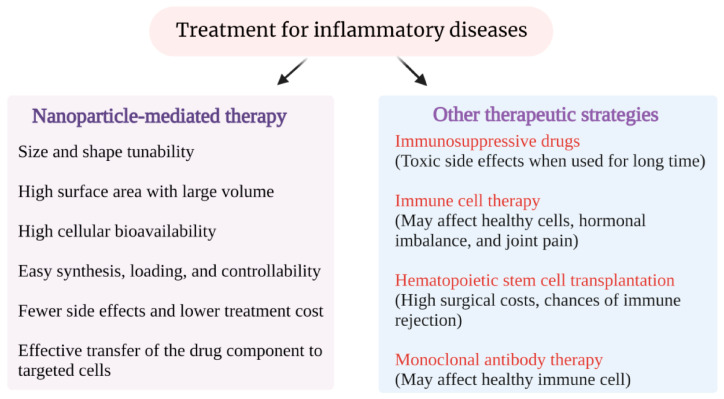
The advantages of NP-mediated therapeutic technologies to treat inflammatory diseases. Reproduced with permission from MDPI, Reference [75].

Various anti-inflammatory drugs can be used by silica aerogel drug delivery systems. Silica aerogel serves as an effective drug carrier for anti-inflammatory drugs, enabling efficient drug loading and controlled release. Sivamaruthi et al. [75] provided a detailed overview of the application of mesoporous silica-based nanoplatforms in the treatment of inflammatory diseases, comparing them with traditional methods to highlight their advantages (Figure 4). These characteristics provide a relevant theoretical basis for silica aerogel drug delivery, which is crucial for effective anti-inflammatory treatment. Caputo et al. studied the adsorption of the anti-inflammatory drug nimesulide under various temperature, pressure, and concentration conditions and fitted it with a model. Silica aerogel doped by the supercritical fluid adsorption method was considered for the rapid release of nimesulide drugs [20]. In vitro dissolution tests revealed a significantly accelerated release rate of nimesulide from the SA composite in comparison to its pure crystalline form. This finding underscores the potential of this system for enhanced drug delivery and improved bioavailability. Ketoprofen’s adsorption into the aerogel was found to significantly enhance its release rate compared to its pure crystalline form. In vitro dissolution tests showed that approximately 50% of the ketoprofen was released within the first 5 min from the aerogel, whereas the pure drug took 120 min to achieve a 45% release rate [76]. This rapid release characteristic makes silica aerogels an attractive option for quick anti-inflammatory action.

Sodium diclofenac loaded in silica aerogel also exhibits a controlled release profile, depending on drug diffusion and erosion of the polymer matrix [42]. This system has the potential to provide sustained anti-inflammatory effects and is suitable for applications in chronic inflammatory conditions. The controlled anti-inflammatory drugs can be tailored to provide both rapid and prolonged anti-inflammatory effects. The loading of drugs on silica aerogels enables their wide application in areas ranging from acute pain management to chronic inflammatory diseases. In contrast to traditional microspheres that often suffer from rapid drug release and inflammatory responses, silica aerogels enable more controlled release and exhibit better mechanical stability.

### 4.2. Cancer Therapy

**Figure 5:**
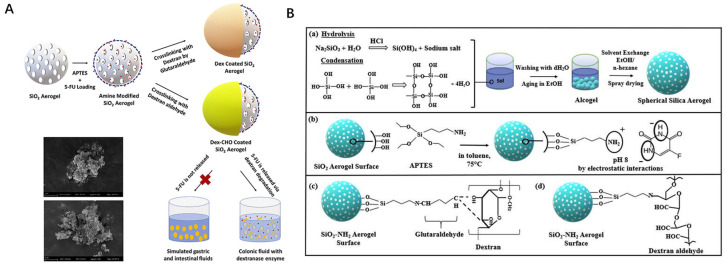
(**A**) Enzyme-triggered and colon-targeted silica aerogel drug delivery system. (**B**) Schematic representation of silica aerogel loaded with 5-FU drug and surface modification. (**a**) synthesis of spherical silica aerogels by sol-gel method, (**b**) functionalization of silica aerogel surface with APTES and loading the 5-FU drug onto aerogel, (**c**) coating of silica aerogel surface with dextran using GA crosslinker, (**d**) coating of silica aerogel surface with dextran aldehyde. Reproduced with permission from Elsevier, Reference [58].

Conventional chemotherapeutic agents have a number of proven shortcomings; they may not effectively reach the tumor site, as well as being less efficient before reaching the target area, and controlling or increasing the dose of the drug often leads to significant side effects [77]. Silica aerogel drug delivery systems enable the oral administration of anticancer drugs, and is inexpensive and easy to produce. Wang et al. prepared silica–gelatin–MTX hybrid aerogels using a co-gelation method, in which the anticancer drug methotrexate could be covalently linked to the collagen molecules of gelatin to improve the drug loading rate. In vitro experiments have shown that the aerogel DDS incubates the aerogel particles triggered by collagenase from tumor cells to produce free methotrexate, with unprecedented cytotoxicity to tumor cells [25]. In order to fully ensure safety for health, a silica–gelatin aerogel delivery system for anticancer drugs was marked with fluorescein to study the biocompatibility and biodistribution pathways after intraperitoneal injection of the particulate suspension in healthy mice. The results showed that histological studies of abdominal organs (liver, spleen, kidneys, thymus) and lymphatic tissues showed no signs of toxicity and that the aerogel particles entered the lymphatic circulation [78]. Aiming at colon cancer, Tiryaki et al. [58] developed an enzyme-triggered drug delivery system. 5-Fluorouracil (5-FU) is a pyrimidine analogue and has been widely used as an antitumor agent. Due to the short half-life of 5-FU, repeated administration is necessary during treatment, which makes it both expensive and inconvenient. Dextran and dextran aldehyde-coated silica aerogel were synthesized, and 5-FU drug was loaded into the modified silica aerogel with 61.2% loading capacity. The rate of release in gastric, intestinal, and enzyme fluids was simulated in vitro, which demonstrated that it was not affected by the gastrointestinal system, and was successful in targeting the colonic region. Figure 5 clearly illustrates the modification process of silica aerogel loaded with 5-FU drug and its drug delivery system. Camptothecin is an anticancer drug with anti-tumor activity, but the activity of CPT is highly pH-dependent and hydrophobic. Follmann et al. used modified nanofibrous silica aerogel particles with highly entangled structural connections between PVA and PAA for the delivery of CPT and showed significant anti-tumor activity against cancer cells in in vitro experiments [56].

### 4.3. Transdermal Therapy

**Figure 6:**
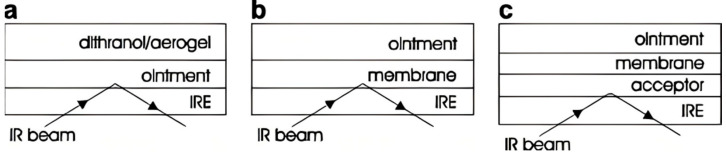
Transdermal experiment of hydrophilic silica aerogels loaded with dithranol: (**a**) release of the drug into the vehicle; (**b**) penetration into the dodecanol collodion membrane; (**c**) penetration in the Nephrophan^®^ membrane or human stratum corneum. Reproduced with permission from Elsevier, Reference [59].

In addition to oral drug delivery system, research on transdermal DDSs is gaining attention. When applied topically, the natural barrier skin restricts the penetration of some drugs. Hence there is a need to design a suitable drug delivery carrier that penetrates the skin barrier, in which aerogels have become the latest technology for topical and transdermal drug delivery [79,80]. Most of the current research emphasizes the use of bio-based aerogels in transdermal drug delivery, like sometimes loading antibiotics. But a few studies of silica aerogel delivery of dermal drug can also be retrieved. In the preparation of hydrophilic silica aerogels, adsorption from the supercritical gas method allows drugs to be uniformly distributed at the molecular level in a non-crystalline state within the highly porous aerogel matrix. The model drug dithranol is used in the treatment of psoriasis vulgaris, and in most of the drugs dithranol is unable to penetrate the stratum corneum without reaching the deeper dermal areas. Guenther et al. studied the permeation of dithranol in silica aerogel carriers to different artificial membranes and human subcutaneous injections (Figure 6), enabling higher fluxes and shorter lag times [59].

**Figure 7:**
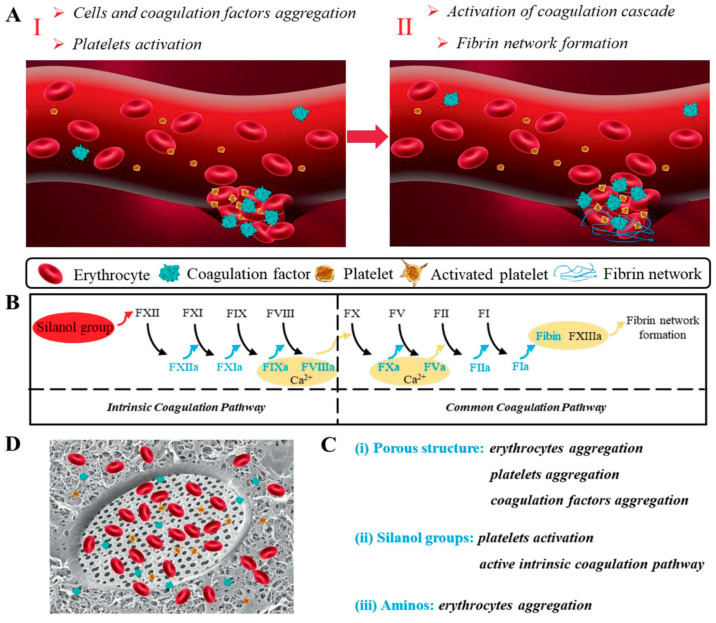
(**A**) Formation of clot at the site of vascular wound after applying CDDs–TBA. (**B**) The potential haemostasis mechanisms of CDDs–TBA. (**C**) Aggregation and activation of erythrocytes, platelets, and coagulation factors on the surface of CDDs–TBA. (**D**) Mechanism of silanol group activates intrinsic coagulation pathway to initiate coagulation cascade. Abbreviation: CDDs: a haemostatic composite material composed of chitosan, dopamine (DA), and diatom–biosilica (DB); TBA: tert-butyl alcohol. Reproduced with permission from Wiley, Reference [81].

Skin wounds often occur in everyday life, and traditional gauze and dressings are constantly being updated, especially in response to uncontrolled bleeding. Silica aerogel synthesized from rice husk ash has good biocompatibility, high level of cellular activity, and cell proliferation for human dermal fibroblasts, which promotes the skin repair process, indicating that SA is suitable for wound care applications [37]. Chitosan with haemostatic properties and a diatom–biosilica matrix as a rapid haemostatic agent have a great potential for application [81]. Figure 7 demonstrates the haemostatic mechanism of silica aerogel composites at the site of vascular wounds. Existing haemostatic agents or dressings have poor haemostatic performance as they impair the function of platelets and weaken the activity of coagulation factors under the harsh conditions of extreme heat and cold, respectively. Silica aerogels can offer unique advantages in haemostatic dressings due to their lower thermal conductivity and density. It was shown that combining hydrophobic nanosilica aerogels with phase change materials could enable them to maintain proper temperature in extremely cold or hot environments by blocking heat conduction and reducing energy loss; their high porosity and large specific surface area and hydrophobicity exhibited excellent haemostatic properties. This study is not only of broad significance for controlling haemorrhage under extremely hot/cold conditions, but extends the application of silica aerogels in wound dressings and opens up a new field of silica aerogel applications [82]. Compared with traditional antibacterial hydrogels or polymeric biomaterials, aerogels offer higher surface-to-volume ratios, enabling greater antimicrobial efficiency with lower dosages.

### 4.4. Bone-Tissue Engineering

**Figure 8:**
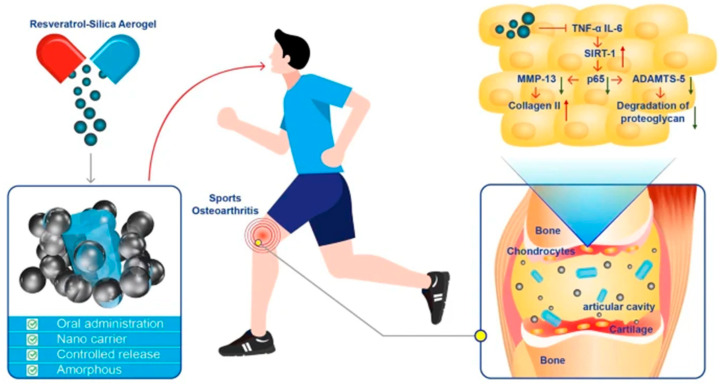
A resveratrol–silica aerogel (RSA) nanodrug complex system enhances the treatment of sports osteoarthritis by activating SIRT-1 through oral administration. Reproduced with permission from Springer, Reference [16].

The silanol-rich groups on the surface of silica aerogels are able to interact with physiological fluids to form an apatite layer similar to that of natural bone. These bioactive properties suggest that silica aerogels can also be used for bone tissue regeneration [35]. The traditional scaffold material polycaprolactone (PCL), a biodegradable polyester, is a good biomaterial for cartilage tissue engineering, but can produce an acidic environment during degradation, which can affect the local microenvironment and cell viability. The acidic conditions can be neutralized by the addition of alkaline silica aerogel during extended incubation periods, contributing to maintained and stimulated cell survival and growth. Although the silica precursors (TEOS and MTMS) of the silica aerogels were different, the composite scaffolds hold good promise for future applications in bone tissue engineering [15,83]. Qin et al. [16] designed a resveratrol-loaded silica aerogel (RSA) as a drug delivery system, which improved the performance of pure resveratrol and demonstrated a sustained release effect. Figure 8 illustrates the therapeutic mechanism of this drug in enhancing the treatment of exercise-induced osteoarthritis. In addition, bone tissue scaffolding materials prepared by mixing silica aerogels with other flexible, bio-based materials, such as poly (lactic acid)/gelatin (PLA/gel) nanofibers [84] and silk fibroin [85], can act as a biologically active porous cellular matrix that fits the defect site and induces both osteogenesis and angiogenesis during the healing of bone defects. Unlike rigid scaffolds that cannot degrade or polymer scaffolds that lack mechanical strength, drug-loaded aerogels provide robust structural stability and tunable degradation rates.

### 4.5. Photothermal Therapy

**Figure 9:**
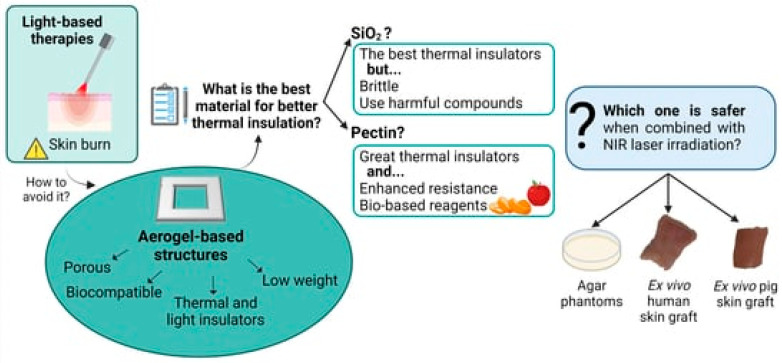
The application of aerogels in photothermal therapy as demonstrated in the abstract. Reproduced with permission from MDPI, Reference [88].

Aerogels are attractive for biomedical applications, with potential use in photothermal therapy (PTT) [86]. The exposure of healthy areas should be reduced to minimize the side effects of this technique on surrounding tissues [87]. Ferreira-Gonçalves et al. investigated silica aerogel as a material for blocking radiation and generated heat in PTT. The thermal protection of silica aerogels under a near-infrared laser irradiation experiment was performed on simulated tissues and in vitro implants. The results show that silica aerogel effectively reduces the temperature increase in the laser-irradiated area compared to the area not covered with aerogel, thus protecting the surrounding healthy tissues from thermal damage. In addition, the silica aerogel showed a good safety profile in skin compatibility tests on human volunteers and did not cause any side effects. These properties suggest that silica aerogel has the potential to be used as a defining material for heat and light in PTT in order to improve treatment efficacy and reduce side effects [88]. Figure 9 summarizes the potential applications of aerogels in photothermal therapy. Wei et al. [60] prepared a multifunctional nanoparticle with a core of mesoporous silica nanoparticles loaded with doxorubicin and a shell of sub-6 nm CuS nanoparticles for combined photothermal and chemotherapy treatment. Compared with conventional metal nanomaterials used for photothermal therapy, aerogels offer unique advantages in porosity and multifunctionalization. Yet, these applications remain largely exploratory.

### 4.6. Gene Therapy

**Figure 10:**
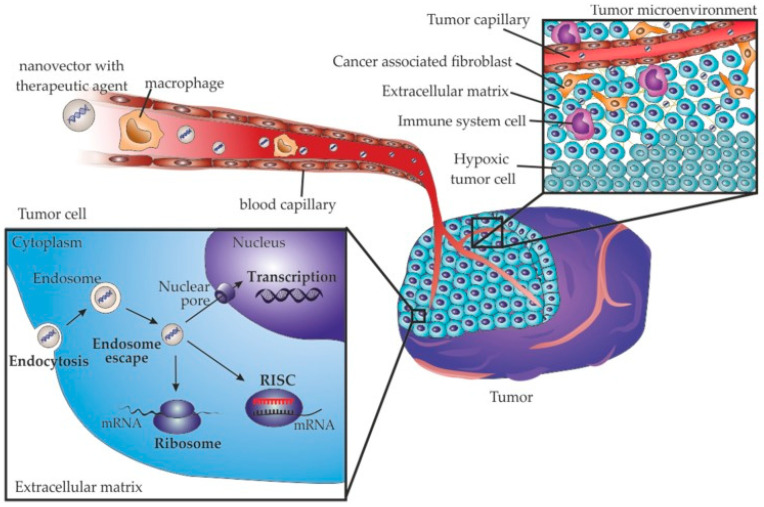
Mechanism diagram of nanoparticle delivery of cancer gene therapy. Reproduced with permission from MDPI, Reference [89].

Gene therapy is the treatment of a genetic disease by the introduction of specific cell function-altering genetic material into a patient. Different from the traditional release of drugs and protein delivery carriers, gene therapy can only be achieved when the required genes are successfully introduced into the target cells. Due to the unique properties of aerogels, gene therapy allows for loading and carrying fragile genes and nucleotides encapsulated and delivered to specific cells. Gene delivery is a promising disease strategy that offers solutions to intractable diseases, such as cancer [89,90]. Figure 10 presents the mechanistic process of cancer gene therapy via nanoparticle delivery. Yetgin and Balkose prepared silica aerogels with high pore volume and surface area for the adsorption of calf thymus DNA and characterized the biosorption process of calf thymus DNA. It was shown that the adsorption of highly polymerized calf thymus self-assembled DNA in the aqueous phase occurs at its macropores and outer surface; hence, silica aerogel is a very promising material for DNA adsorption and provides prospects for the research and application of silica aerogel materials in DNA delivery systems for gene therapy [91].

### 4.7. Pharmaceutical-Containing Wastewater

The exceptional performance of aerogel in drug release can be attributed to its inherent drug adsorption capacity, which allows for a controlled and sustained release of therapeutic agents. This characteristic has not only made it a promising material in the pharmaceutical sector but has also directed attention towards its potential applications in environmental remediation [92,93,94]. Therefore, the application of silica aerogel in removing pollutants from wastewater has also received attention. Wastewater generated in the pharmaceutical process, such as urine and excreta excreted by the patients taking chemotherapy drugs, need sewage treatment. Modified silica aerogels are used as an adsorbent at a relatively low cost and is suitable for the removal of chemotherapy drugs from the wastewater [95,96]. Ghahremani et al. [97] successfully synthesized a novel modified silica aerogel (NMSA) via the sol–gel method, which exhibited a maximum Pb (II) removal efficiency of 75% at pH 4 after process optimization. Alyne Lamy-Mendes et al. [98] discovered that the presence of amine groups in silica aerogels/xerogels significantly enhances their potential as alternative industrial sorbents, enabling high and rapid removal efficiency towards various pollutants. Lamy-Mendez et al. [99] also investigated the removal capacities of carbon nanomaterials–MTMS-based silica aerogel for various contaminants in aqueous solutions. They found that adding carbon nanotubes increases the removal efficiency by more than 71% for amoxicillin and 96% for naproxen. These novel materials demonstrate the potential of silica-based composite aerogels in the treatment of pharmaceutical wastewater.

## 5. Biomedical Safety and Toxicity

### 5.1. In Vitro and In Vivo Biocompatibility

In Vitro Biocompatibility. The polyurea-coated silica aerogel prepared by Keller et al. exhibited good biocompatibility and low toxicity at low doses. However, at high doses (>0.6 mg/lung), it may induce a mild inflammatory response, manifested by an increase in specific enzyme activity, which indicates damage to cells or lysosomes [100]. Pavan et al. [101] found that silica particles with a population of nearly free silanols damage cellular membranes and initiate inflammatory reactions. Moreover, the surface properties of silica aerogels play a significant role in their biocompatibility and potential toxicity. The surface chemistry of silica aerogel affects protein adsorption, cell uptake, and subsequent immune responses.

In Vivo Biocompatibility. In vivo studies are essential to evaluate the long-term safety and effects of silica aerogels in biological systems. Currently, the majority of in vivo studies of silica aerogels have primarily focused on biocompatibility in animal models. Kortesuo et al. [102] implanted silica xerogels loaded with toremifene into the subcutaneous tissue of mice for 42 days. The xerogels exhibited sustained release in vivo, with a residual amount of approximately 16%, and no pathological changes were observed in organs. Reyes-Peces et al. [103] implanted gelatin-hybridized silica aerogels into rabbit femoral defects, demonstrating low immunogenicity. The aerogels were degraded by lysosomal enzymes, with a degradation rate that matched the rate of bone regeneration, thereby avoiding material residue.

### 5.2. Cytotoxicity and Biodegradability

The porous structure and adjustable surface properties of silica aerogel enable it to be effectively phagocytosed by cells after delivering drugs in vivo. The charge and hydrophilicity of the silica aerogel surface affect the efficiency of cell phagocytosis. The charge of nanoparticles (NPs) determines the blood circulation time, uptake rate, and the intended target cells. Research has shown that negatively charged NPs can be taken up by cells through various endocytosis pathways, such as clathrin-mediated endocytosis and caveolae-mediated endocytosis [104]. However, due to differences in cell types, negatively charged NPs may be difficult to uptake due to excessive aggregation, such as in human osteosarcoma cells (MG63) [105]. The hydrophilicity primarily influences the drug release mechanism after cells phagocytose the silica aerogel. Hydrophilic silica aerogels can rapidly release drugs in the intracellular environment, while hydrophobic aerogels can achieve a longer-lasting slow-release effect [64].

In addition to cytotoxicity, the biodegradability of silica aerogels is also a crucial determinant of their long-term safety for biomedical applications. Silica-based nanomaterials can gradually degrade in physiological conditions into soluble orthosilicic acid (Si(OH)_4_), which is naturally excreted through the kidneys. Smaller and highly porous aerogels degrade faster, whereas surface modifications, such as PEGylation, can slow down dissolution and prolong circulation time [106]. Future research should focus on striking a balance between controlled degradation and sufficient stability, aiming to achieve sustained drug release while avoiding unwanted persistence or accumulation in vivo.

### 5.3. Immune and Inflammatory Response

During the phagocytosis process, silica aerogel may induce immune regulation and inflammatory responses. When macrophages attempt to phagocytose large-sized aerogels, the cells partially cover the particles and activate immune cells, causing a frustrated phagocytosis phenomenon, which in turn triggers an inflammatory response. In addition to frustrated phagocytosis, aerogel particles can also induce cell surface reactions, promoting the release of soluble molecules, such as cytokine secretion, ultimately contributing to the onset of inflammation [107]. The design of conventional silica aerogel DDSs can be optimized through modification of the surface properties of silica aerogel to regulate its interaction with macrophages and reduce inflammatory responses. From another perspective, future research can be based on this characteristic to develop novel silica aerogel drug delivery systems for the treatment of inflammatory and infectious diseases. Beyond phagocytosis, other immune cells, such as dendritic cells and T lymphocytes, may also interact with silica aerogels, influencing both innate and adaptive immune responses [108]. These findings suggest that the immunological profile of silica aerogels can be finely tuned, which can leverage immune activation for therapeutic purposes.

### 5.4. Potential Long-Term Safety Issues

Moderate amounts of silica aerogel can be degraded in lysosomes within cells without causing significant toxicity to the cells. Although silica aerogels generally exhibit good biocompatibility and low toxicity, the potential toxicity of high doses of drug delivery during the cell phagocytosis process still needs to be considered. Most studies have focused on short-term exposure, with few studies evaluating the effects beyond several weeks, which means chronic toxicity data on drug-loaded aerogels are relatively scarce [109]. High doses of silica aerogels can lead to tissue inflammation and organ damage. Dose–response relationships are not well established, particularly regarding accumulation in sensitive organs, such as the liver, spleen, and kidneys [110]. Addressing these gaps will require long-term animal studies, standardized safety assessment protocols, and early alignment with regulatory frameworks. Therefore, it is necessary to optimize the dose and surface properties of silica aerogels in order to minimize potential toxic effects [111].

## 6. Summary and Future Research Strategies

### 6.1. Integration of Silica Aerogels with Smart Stimuli-Responsive DDSs

**Figure 11:**
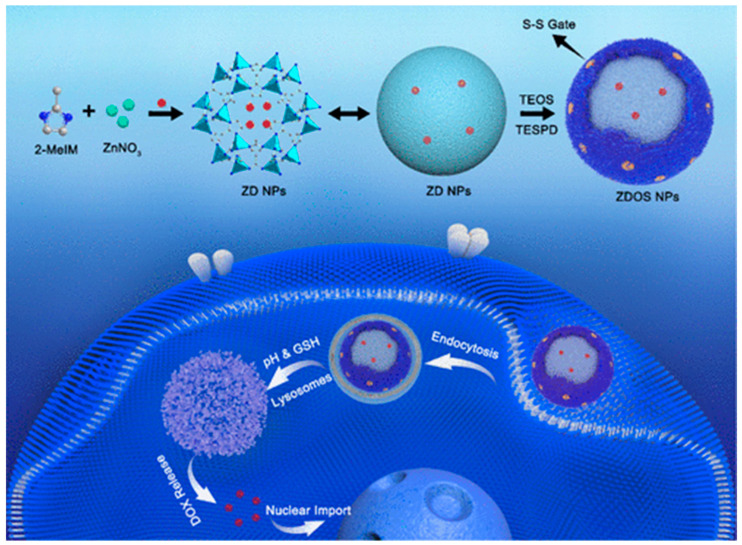
Schematic illustration of the preparation of the ZIF-8@DOX@organosilica (ZDOS) NPs as a pH and redox dual-responsive DDSs for cancer therapy. Reproduced with permission from ACS, Reference [113].

Stimuli-responsive DDSs represent a new generation of carriers that enable site-specific and on-demand drug release, thereby enhancing therapeutic efficacy and reducing systemic side effects, while achieving precise spatiotemporal control over drug delivery. The targeted delivery capabilities of DDSs are primarily driven by pH sensitivity, ligand–receptor interactions, morphology control, and surface modifications [112]. Figure 11 shows a schematic diagram of the preparation and action of organic silica nanoparticles used in cancer treatment.

(1) pH sensitivity

Currently, pH-sensitive smart polymer materials are mostly used in the field of hydrogels. Given the significant pH variations inherent in human pathophysiological microenvironments, they show significant application potential in targeted drug delivery systems [114]. Zhao et al. [115] prepared a CSPBA/PVA/OHC–PEG–CHO hydrogel. Under pH 7.4 conditions and in the presence of glucose. Drug release was significantly better than under other conditions alone (pH 7.4 or pH 6.5). This dual-response mechanism is particularly suitable for diabetic wounds. Alnaief et al. used spouted bed technology to achieve a double-layer coating of silica aerogel particles loaded with ibuprofen using PEG 2000 and pH-sensitive polymer Eudragit^®^ L [116]. The stability of the polymers varies with the pH. Eudragit^®^ L dissolves at pH values over 5.5, and the coating does not affect the release rate of ibuprofen under neutral pH conditions.

(2) Ligand–receptor interactions

In cancer treatment, the most widely used method is to deliver drugs to the tumor cells by wrapping or combining them with specific carriers that bind specifically to target surface antigens or receptors [117]. By controlling the synthesis process, silica aerogels can be fabricated into various shapes, such as nanoparticles, fibers, microspheres, and designed three-dimensional structures with distinct targeting properties [118]. Generally speaking, smaller nanoparticles are more readily internalized by cells, and are commonly used as intracellular drug delivery vehicles.

(3) Surface modifications

Specific surface modifications can further enhance the interaction between silica aerogel and cell membranes, achieving targeted delivery. By modifying the surface of silica aerogel with bioactive molecules not only improved the efficiency of cell uptake of drug-loaded aerogel but enhanced the delivery effect of the drug by regulating the intracellular signaling pathways [119,120]. Tiryaki et al. [58] modified the surface of silica aerogel with dextran (Dex) and dextran aldehyde (Dex-CHO), and then loaded 5-fluorouracil onto the aerogel. The design utilizes the dextranase present in the colon to trigger drug release, thereby achieving colon-targeted drug delivery.

In addition to post-synthesis modification, in situ functionalization also plays a critical role. In situ functionalization refers to the introduction of functional components during the sol–gel process of silica aerogels. This approach can avoid damage to the porous structure caused by post-synthesis modification. Mi Mi Wan et al. [121] employed in situ drug loading to incorporate a hydrophilic drug (heparin) and a hydrophobic drug (ibuprofen) into silica aerogels, thereby reducing preparation time and enhancing both drug loading and release. Additionally, silica aerogels can also be integrated with thermo-responsive polymers, photothermal agents, magnetic nanoparticles, and enzyme-sensitive linkers, offering versatile platforms for various medical applications.

### 6.2. Hybrid Systems to Improve Pharmacokinetics and Targeting

In current research, the drug loading design for silica aerogel has been achieved, and its release effect in drug delivery has been proven. The highly porous structure of silica aerogel provides an excellent platform for loading various types of drugs. By modifying the preparation conditions, silica aerogels with different pore size distributions and surface properties can be prepared, enabling controlled drug release [122,123]. Drugs with different solubility and stability can be introduced at different stages of the sol–gel process, thereby enabling the loading of multiple drugs. Based on the material properties of silica aerogels, their pharmacokinetic (PK) performance and targeting ability can be further enhanced by hybridization with other functional materials. Hybrid systems combine the structural advantages of aerogels with the complementary properties of other components, creating multifunctional carriers for controlled release. It is expected that multiple drugs can be loaded and drug release controlled in future research.

(1) Improving pharmacokinetics

**Figure 12:**
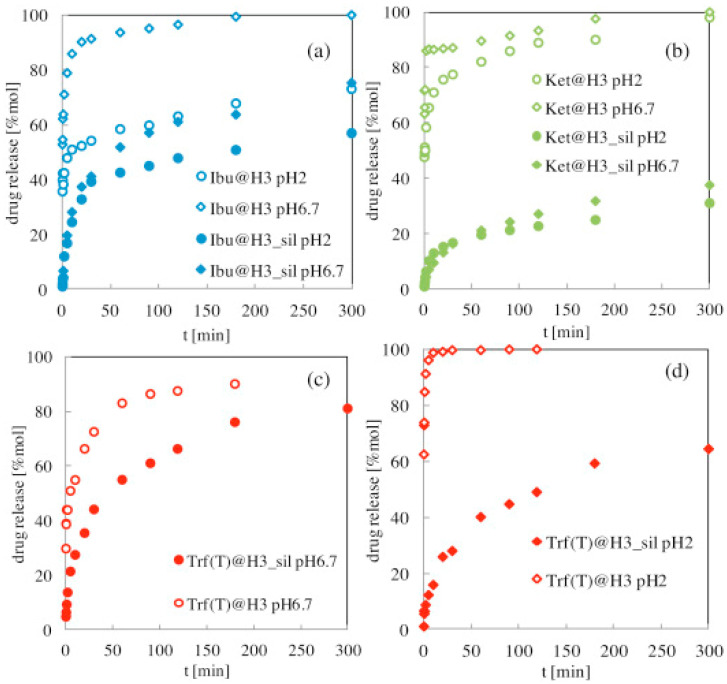
Drug release profiles of ibuprofen (Ibu), ketoprofen (Ket), and triflusal (Trf) from hybrid silica/gelatin (3 wt%) aerogels without hydrophobic groups (H3) or with silylated groups (H3_sil) at pH 6.7 and 2. (**a**) ibuprofen at pH 6.7 and 2, (**b**) ketoprofen at pH 6.7 and 2, (**c**) triflusal at pH 6.7, and (**d**) triflusal at pH 2. Reproduced with permission from Elsevier, Reference [125].

In the development of silica aerogel preparation, modification of silica aerogels often leads to enhancement of drug loading and controlled release capabilities, making practicality possible. Smirnova et al. [41] investigated the loading of several poorly water-soluble drugs into silica aerogels using scCO_2_. They reported high loadings of ketoprofen (30 wt%), miconazole (60 wt%), and ibuprofen (73 wt%) from their respective saturated solutions, attributed to their high solubility in the scCO_2_ phase. After surface modification of the aerogels with different functional groups, the release kinetics of drugs can be further regulated [124]. Figure 12 shows the different release curves of the three drugs at 37 °C under pH conditions of 2 and 6.7 [125]. Hydrophobic-modified silica/gelatin aerogel showed more sustained release, particularly at pH 2, due to a strengthening between the drug and the matrix through hydrogen bonds. Ganonyan et al. [55] developed a progressive hydrophobic process for silica aerogel, which was modified with trimethylchlorosilane vapor for varying durations. By preventing the collapsing of the aerogel pores, the release kinetics of a model drug can be increased or decreased by controlling the hydrophobization process.

(2) Enhancing targeting

Hybridization also allows silica aerogels to be tailored for tissue-specific or cellular targeting. Targeting of aerogel-based carriers can be achieved via both passive and active mechanisms Passive targeting relies principally on the enhanced permeability and retention (EPR) effect in solid tumors, which allows aerogels to extravasate and accumulate in tumor interstitium [126]. Active targeting improves tissue or cellular selectivity by decorating carrier surfaces with ligands which bind receptors overexpressed on target cells. Studies in silica-based carriers show that ligand conjugation can increase cell-specific uptake and reduce off-target accumulation, but the benefit depends on ligand density, orientation, and the biological accessibility of the target receptor [127]. In addition, hybridization also provides extra ways to target. Silica–photothermal hybrids enable localized heating under NIR light to trigger release and to produce synergistic chemo-photothermal therapy, which enhances intratumoral delivery and therapeutic efficacy [128]. However, it is worth noting that, while surface functionalization can enhance targeting, hybrid payloads may also alter biodegradation and safety profiles.

### 6.3. Scale-Up Challenges for Industrial and Clinical Translation

(1) Large-scale production and clinical translation

Despite the promising laboratory-scale results, the large-scale production and clinical translation of silica aerogels still remain challenging. One of the primary barriers is the high manufacturing cost and the complexity of the production process. Although supercritical drying technology can produce high-quality aerogels, its requirement for high-pressure conditions and substantial energy consumption restricts large-scale production. While freeze-drying has lower pressure requirements, it is similarly time-consuming and costly. By contrast, ambient pressure drying is the most cost-effective option. However, it often leads to pore collapse and shrinkage, which can damage the aerogel structure and properties, thereby significantly affecting drug delivery efficacy. Future exploration of additional surface modification steps for drug-loaded aerogels is crucial for maintaining aerogel structure and enabling large-scale production.

(2) Reproducibility of Production

Another challenge is the reproducibility of production. During the preparation of aerogels, variations in the precursor solution, the sol–gel process, and the drying conditions can significantly influence the aerogel’s pore structure and drug-loading capacity. The incorporation of sensitive biomolecules or drugs into aerogels introduces issues related to drug stability and uniform distribution, which are even more challenging to maintain in large-scale batches. For biomedical applications, strict quality control, sterility assurance, and validated reproducibility are also required to meet pharmaceutical standards. Overcoming these limitations requires the development of simplified, energy-efficient, and standardized processes to ensure the structural stability and reproducibility of drug-loaded aerogels, thereby facilitating the transition of silica aerogels from laboratory research to clinical-grade production.

### 6.4. Regulatory Pathways for Approval of Aerogel-Based Carriers

Before being marketed, drug-loaded aerogels necessitate a comprehensive evaluation of biocompatibility, biodegradability, pharmacokinetics, immunogenicity, and long-term safety, supported by robust evidence from both in vitro and in vivo studies. To achieve clinical adoption, aerogel-based drug delivery systems need to navigate established regulatory frameworks, such as the Center for Drug Evaluation and Research (CDER) of the U.S. Food and Drug Administration (FDA) and the Center for Drug Evaluation (CDE) of the China National Medical Products Administration (NMPA). In addition, the manufacturing process of drug-loaded aerogels must comply with the Good Manufacturing Practice (GMP) standards of the World Health Organization (WHO) to ensure product consistency and sterility.

Silicon dioxide has been recognized by the FDA as a material that is “generally recognized as safe” (GRAS) for use in animal feed [129]. Currently, silica aerogels remain a relatively novel class of carriers, and there are no clear clinical analogues that have been approved by drug regulatory authorities. Therefore, the approval process may vary on a case-by-case basis, necessitating close communication with regulatory agencies. Collaborating with regulatory agencies early in the development process to understand and meet all necessary standards is essential. Meanwhile, implementing processes that comply with the GMP standards and conducting thorough validation studies can help ensure drugs meet market requirements.

## 7. Conclusions

Presently, nanocarrier systems are a popular trend for drug delivery. Silica aerogels are superior to other nanomaterials due to their porous, low density, simple, and modifiable structure, and have received much attention in the field of drug delivery due to their proven preparation process and safety benefits. This review systematically summarizing the intrinsic advantages of silica aerogels and justifying their uniqueness as a “multifunctional, low-risk” drug delivery system. The strengths make them a platform for the delivery of drugs and bioactive ingredients. Functionalizing its hydrophilicity allows the delivery of both water-soluble and insoluble drugs, and the silica-based hybrid aerogels have even better advantage of mechanical properties, biocompatibility, and targeted release.

This paper reviews recent routine applications of silica aerogels for the delivery of different types of drugs (e.g., anti-inflammatory, anticancer, transdermal drugs, etc.). Additionally, this review extends the application scope of silica aerogels beyond drug delivery to include emerging fields such as gene therapy and pharmaceutical wastewater remediation. By comparatively analyzing preparation techniques, critically evaluating drug-loading strategies, and highlighting regulatory and translational challenges, we present a comprehensive framework for advancing silica aerogel-based carriers toward biomedical applications. The use of silica aerogels as carriers can have treatment benefits as well as the conditions for batch preparation, with a research basis for various in vitro and in vivo therapies. In future, through continued clinical studies on silica aerogels drug delivery systems, coupled with the optimization of hybrid structures and targeting strategies, it is expected to overcome existing challenges (e.g., rapid clearance in vivo, controlled drug release). This will facilitate the widespread clinical application of silica aerogels as nanocarriers and bring new opportunities for personalized medicine and the environmental remediation of pharmaceutical pollutants.

## Figures and Tables

**Table 1:** Critical evaluation of drug-loading routes for silica aerogels.

Loading Route	Advantages	Limitations	Suitability for Biomedical Applications
Sol–gel process	Simple, direct incorporation of drugs; uniform distribution of drugs	Limited to drugs stable under sol–gel conditions; risk of denaturation for biomolecules	Suitable for small-molecule and stable hydrophilic drugs
Aging process	Without additional chemical steps; allows drugs to diffuse into pores	Dependent on drug solubility in the aging solvent	Suitable for soluble drugs with acceptable stability in solvent
In the dried aerogel	Preserves drug activity; allows the loading of drugs with various solubilities.	Needs additional reagents and extra time	Suitable for poorly water-soluble drugs; enhancing bioavailability
scCO_2_ adsorption	Without organic solvent residues; uniform distribution of poorly soluble drugs	Limited to CO_2_-soluble drugs, requires high-pressure equipment	Suitable for hydrophobic or poorly water-soluble drugs
Solvent-based impregnation	Allows a variety of drug loading; relatively simple;	Potential of residual solvents; simple operation	Widely applicable, but requires to meet (ICH) Q3 (R5) guideline
Surface functionalization	Enables targeted delivery; controls drug release	Involves additional chemical steps and reagents; may alter biocompatibility	Promising for controlled release and targeted therapy

## Data Availability

No new data were created or analyzed in this study.
